# Bacterial symbionts of the leafhopper *Evacanthus interruptus* (Linnaeus, 1758) (Insecta, Hemiptera, Cicadellidae: Evacanthinae)

**DOI:** 10.1007/s00709-015-0817-2

**Published:** 2015-04-22

**Authors:** Teresa Szklarzewicz, Beata Grzywacz, Jacek Szwedo, Anna Michalik

**Affiliations:** Department of Developmental Biology and Morphology of Invertebrates, Institute of Zoology, Jagiellonian University, Gronostajowa 9, 30-387 Kraków, Poland; Institute of Systematics and Evolution of Animals, Polish Academy of Sciences, Sławkowska 17, 31-016 Kraków, Poland; Department of Invertebrate Zoology and Parasitology, Faculty of Biology, University of Gdańsk, Wita Stwosza 59, 80-308 Gdańsk, Poland

**Keywords:** Symbiotic microorganisms, *Sulcia*, Bacteriocytes, Transovarial transmission of symbionts, Cicadellidae

## Abstract

Plant sap-feeding hemipterans harbor obligate symbiotic microorganisms which are responsible for the synthesis of amino acids missing in their diet. In this study, we characterized the obligate symbionts hosted in the body of the xylem-feeding leafhopper *Evacanthus interruptus* (Cicadellidae: Evacanthinae: Evacanthini) by means of histological, ultrastructural and molecular methods. We observed that *E. interruptus* is associated with two types of symbiotic microorganisms: bacterium ‘*Candidatus* Sulcia muelleri’ (Bacteroidetes) and betaproteobacterium that is closely related to symbionts which reside in two other Cicadellidae representatives: *Pagaronia tredecimpunctata* (Evacanthinae: Pagaronini) and *Hylaius oregonensis* (Bathysmatophorinae: Bathysmatophorini). Both symbionts are harbored in their own bacteriocytes which are localized between the body wall and ovaries. In *E. interruptus*, both *Sulcia* and betaproteobacterial symbionts are transovarially transmitted from one generation to the next. In the mature female, symbionts leave the bacteriocytes and gather around the posterior pole of the terminal oocytes. Then, they gradually pass through the cytoplasm of follicular cells surrounding the posterior pole of the oocyte and enter the space between them and the oocyte. The bacteria accumulate in the deep depression of the oolemma and form a characteristic ‘symbiont ball’. In the light of the results obtained, the phylogenetic relationships within modern Cicadomorpha and some Cicadellidae subfamilies are discussed.

## Introduction

The Hemiptera are a large group of insects with feeding habits that range from phytophagy to predation, including ectoparasitism and hematophagy (Forero 2008). Plant feeders suck the phloem or xylem sap, or ingest plant cell content (Backus 1988; Campbell et al. 1994; Sorensen et al. 1995). Among the six suborders of the Hemiptera (i.e. Paleorrhyncha,Sternorrhyncha, Fulgoromorpha, Cicadomorpha, Coleorrhyncha and Heteroptera; Szwedo et al. 2004; Grimaldi and Engel 2005; Drohojowska et al. 2013), the Cicadomorpha: Clypeata lineage (uniting extinct Hylicelloidea and extant Cicadoidea, Cercopoidea and Membracoidea *sensu lato*) is the only one with several strong adaptations for xylem feeding (Wang et al. 2012), however in some of them shifts to cell-content and back to phloem feeding occurred (Dietrich 2013). The diet of xylem-feeding hemipterans is extremely unbalanced, because the amount of nutrients in the xylem sap is 10 times less than that in the phloem (Andersen et al. 1989). Therefore, most plant sup-sucking hemipterans live in mutualistic associations with symbiotic microorganisms (bacteria or yeast) which synthesize missing nutrients and provide them to their hosts (Wilkinson and Ishikawa 2001; Baumann 2005; Douglas 2009). The sequencing of the genome of bacteria inhabiting the body of hemipterans has indicated that these microorganisms possess the biosynthetic pathways necessary for the synthesis of essential amino acids, i.e. leucine, serine, tryptophan as well as many vitamins and other cofactors (Douglas 2006; Wu et al. 2006; McCutcheon and Moran 2007; McCutcheon et al. 2009).

The obligate symbionts may be localized both extracellularly, in the lumen of midgut appendages (in some heteropterans), and intracellularly, in cells of the midgut epithelium (in some heteropterans) or in specialized cells of mesodermal origin termed bacteriocytes/mycetocytes (in most hemipterans) (see Kikuchi 2009 for further details). The latter microorganisms are termed ‘mycetomic symbionts’.

Among hemipterans, the Sternorrhyncha (aphids, psyllids, whiteflies, scale insects) are associated with one type of obligate mycetomic symbiont (termed ‘primary symbiont’), e.g. aphids harbor the bacterium *Buchnera aphidicola*, psyllids – the bacterium *Carsonella ruddii* (see Baumann 2005 for further details). Apart from obligate symbionts, sternorrhynchans, as a rule, hold additional symbionts (termed secondary or facultative symbionts) that may play various roles for their host insects, e.g. may protect them from infection by fungal pathogens or attack by parasitic hymenopterans (Oliver et al. 2003; Scarborough et al. 2005). In contrast to the situation observed in Sternorrhyncha, in Cicadomorpha and Fulgoromorpha (both formerly treated as Auchenorrhyncha), two or more types of obligate symbionts (termed ‘coprimary symbionts’) co-occur and all of them are engaged in the synthesis of nutrients essential to the host insect (Moran et al. 2003; Takiya et al. 2006; Bressan et al. 2009; Noda et al. 2012; Urban and Cryan 2012; Bennett and Moran 2013; Ishii et al. 2013; Koga et al. 2013; Michalik et al. 2014a; Wu et al. 2006).

Molecular analyses have shown that both Cicadomorpha and Fulgoromorpha usually possess the obligate Bacteroidetes bacterium ‘*Candidatus* Sulcia muelleri’ (hereafter referred to as *Sulcia*) and one other type of coprimary symbiont, e.g. gammaproteobacterium ‘*Candidatus* Baumannia cicadellinicola’ (hereafter *Baumannia*), betaproteobacterium ‘*Candidatus* Zinderia insecticola’ (hereafter *Zinderia*), betaproteobacterium ‘*Candidatus* Vidania fulgoroideae’ (hereafter *Vidania*), betaproteobacterium ‘*Candidatus* Nasuia deltocephalinicola’ (hereafter *Nasuia*), alphaproteobacterium ‘*Candidatus* Hodgkinia cicadicola’ (hereafter *Hodgkinia*) (Moran et al. 2003, 2005; Takiya et al. 2006; McCutcheon et al. 2009; Gonella et al. 2011; Noda et al. 2012; Urban and Cryan 2012; Ishii et al. 2013; Koga et al. 2013). The results of recent molecular phylogenetic analyses (Bennett and Moran 2013; Koga et al. 2013; Bennett et al. 2014; Koga and Moran 2014) suggest that this enormous diversity of symbionts found in Cicadomorpha and Fulgoromorpha results from a multiple, independent replacement of symbiotic bacteria by other microorganisms. An ancestor of Cicadomorpha and Fulgoromorpha has been colonized by *Sulcia* and betaproteobacterial symbionts (probably 270 million years ago), but during the further evolution of the hemipteran lineages the betaproteobacterial co-symbiont was replaced by other bacteria (Moran et al. 2005; Bennett and Moran 2013; Koga et al. 2013; Koga and Moran 2014). In some planthopper families, i.e. Flatidae and Delphacidae as well as in leafhopper *Scaphoideus titanus* (Cicadellidae: Deltocephalinae) bacterial symbionts have been replaced by yeast symbionts (Sacchi et al. 2008; Michalik et al. 2009; Noda 1977).

On account of their crucial role, the obligate symbionts of hemipterans are transovarially (vertically) transmitted between generations (see Buchner 1965 for further details). For this reason, bacteria living in different species of insects evolved independently from one another, i.e. without gene exchange between bacteria in different hosts.

*Evacanthus interruptus* (Linnaeus, 1758) is a representative of leafhoppers (Cicadomorpha: Membracoidea: Cicadellidae). So far, the symbiotic microorganisms present in the xylem-feeding subfamily Evacanthinae Metcalf, 1939 have been examined only fragmentarily (Buchner 1965; Takiya et al. 2006). Early microscopic observations conducted by Buchner (1965) showed that the females of *Evacanthus interruptus* possess two types of symbiotic microorganisms, which are localized in separate bacteriocytes. More recently, using molecular methods, Takiya et al. (2006) indicated the occurrence of *Sulcia* symbiont in the *Pagaronia tredecimpunctata* species Ball, 1902. Koga et al. (2013) detected betaproteobacterial symbionts in the same leafhopper species.

The objectives of this study were to examine the ultrastructure of symbionts of *E. interruptus*, their distribution in the host insect body, mode of transovarial transmission from one generation to the next and to determine their systematic affinity.

## Material and methods

### Insects

Adult females of *Evacanthus interruptus* (Linnaeus, 1758) were collected from herbaceous plants in Kraków (Poland) and near Vorokhta (Chornohora Mountain, Eastern Carpathians, Ukraine), from July to September.

### DNA analyses

The dissected bacteriomes were fixed in 96 % ethanol, washed twice in sterile water and homogenized in 120 μl of 0.7 M NH_4_OH. After 15 min of incubation at 100 °C in alkaline conditions, tubes were opened and further incubated at 100 °C for 10 min. Next, the samples were centrifuged (5 min, 12 000 rpm) and supernatants were transferred to the new tubes. Two volumes of 96 % ethanol and 1/10 volume of 3 M sodium acetate were added to every tube. DNA precipitations were carried out for 30 min at −20 °C. After centrifugation (15 min, 12 000 rpm, 4 °C), DNA pellets were washed in 250 μl of 70 % ethanol and centrifuged (15 min, 12 000 rpm, room temperature). The DNA was then dried for 15 min at 37 °C, dissolved in 20 μl of sterile water, and stored at −20 °C for further analysis.

The polymerase chain reaction (PCR) for the detection of the symbionts harbored in the bacteriocytes of *E. interruptus* were performed using the following specific primers: 10CFBFF (5’AGAGTTTGATCATGGCTCAGGATG3’) and 1515R (5’GTACGGCTACCTTGTTACGACTTAG3’) for *Sulcia* symbiont (Moran et al. 2005); 16SA1 (5’AGAGTTTGATCMTGGCTCAG3’) and Bet940R (5’TTAATCCACATCATCCACCG3’) (Koga et al. 2013) for betaproteobacterial symbiont, as well as 10 F (5’AGTTTGATCATGGCTCAGATTG3’) and 650R (5’CACCGGTACATATGAAATTCT3’) for *Baumannia* symbiont (Takiya et al. 2006). The PCR mixture contained 2 μl of 10x PCR buffer, 0.3 μl of 25 mM MgCl_2,_ 0.5 μl of 10 mM deoxynucleotide triphosphate (dNTP) mixture, 0.5 μl of each 10 mM forward and reverse primers, 2 μl of 1000 ng/μl extracted DNA, 1.25U of JumpStart™ Taq DNA polymerase (Sigma-Aldrich, Germany) and 12.7 μl of sterile, deionized water. The PCR profile consisted of an initial denaturation step at 94 °C for 3 min, followed by 33 cycles at 94 °C for 30 s, 50 °C (for primers:10 F and 650R), 52 °C  (for primers: 16SA1 and Bet940R) or 55 °C (for primers:10CFBFF and 1515R) for 40 s, 70 °C for 1 min 40 s and a final extension step of 5 min at 72 °C. PCR products were subjected to electrophoresis on 1 % agarose gel stained with ethidium bromide and purified using the GenElute MinusEtBr Spin Columns (Sigma-Aldrich, Germany). The purified amplification products were used for sequencing in two directions. The obtained sequences were deposited in GenBank database with accession numbers KP278014–KP278015 and KP943506–KP943507.

### Phylogenetic analyses

The sequences of the 16S rDNA of symbiotic microorganisms used in comprehensive phylogenetic analyses were downloaded from GenBank. The sequences were edited using BioEdit Sequence Alignment Editor 5.0.9 (Hall 1999), and aligned using ClustalX 1.8 (Thompson et al. 1997). All alignments were verified and corrected visually. For the analyses, the appropriate nucleotide substitution model was first determined using Modeltest 3.06 (Posada and Crandall 1998). Based on fossil evidence (Moran et al. 2005; Bennett and Moran 2013; Koga et al. 2013; Koga and Moran 2014) that calibration points required for the molecular dating analyses were assigned as follows: the Cicadomorpha crown clade was fixed at 270 MYA. Molecular clock analyses were conducted using 16S rDNA. Beast v 1.6.1 (Rambaut and Drummond 2007) was used to infer divergence times of lineages under a Bayesian statistical framework. The time of lineage divergence was estimated using the uncorrelated lognormal relaxed clock model. For each matrix, Beast was run using a Yule speciation process and two independent MCMC chains for 4 000 000 generations, sampling every 100th generation. The convergence to stationary and the effective sample size of the model parameters were checked using Tracer. The maximum clade-credibility trees were built with TreeAnnotator (Rambaut and Drummond 2007). FigTree 1.4.0 software (Rambaut 2008) was used to visualize the results including confidence intervals. Pairwise, genetic distances were computed using Mega 6.0 (Tamura et al. 2013).

### Light and electron microscopy

Entire abdomens, dissected ovaries and bacteriomes destined for histological and ultrastructural studies were fixed in 2.5 % glutaraldehyde in 0.1 M phosphate buffer (pH 7.4) at room temperature. The material was next rinsed in the phosphate buffer with an addition of 5.8 % sucrose and postfixed in 1 % osmium tetroxide (in the same buffer). After undergoing dehydration in a graded series of ethanol and acetone, the material was embedded in epoxy resin Epon 812 (Serva, Germany). Semithin sections (1 μm thick) were stained with 1 % methylene blue in 1 % borax and examined and photographed under light microscopes, Leica DMR and Nikon Eclipse 80i. Ultrathin sections (90 nm thick) were contrasted with lead citrate and uranyl acetate and analyzed and photographed in the Jeol JEM 100 SX and Jeol JEM 2100 (Jeol, Japan) electron microscopes.

### Fluorescence microscopy

The dissected ovaries destined for histochemical studies were fixed in 4 % formaldehyde in phosphate buffered saline (PBS) for 45 min at room temperature, rinsed in the same buffer, dehydrated in a graded series of ethanol, embedded in Histocryl (Agar Scientific LTD, Stansted, UK) and cut into semithin sections (0.7 μm thick). The sections were then stained with DAPI (1 μg/ml; Sigma Chemical Co., St. Louis, USA) for 30 min in darkness at room temperature. Next, the material was examined and photographed under an Axiovert 200 M confocal microscope.

## Results

### Molecular identification and phylogenetic analysis of symbionts of *Evacanthus interruptus*

**Fig. 1:**
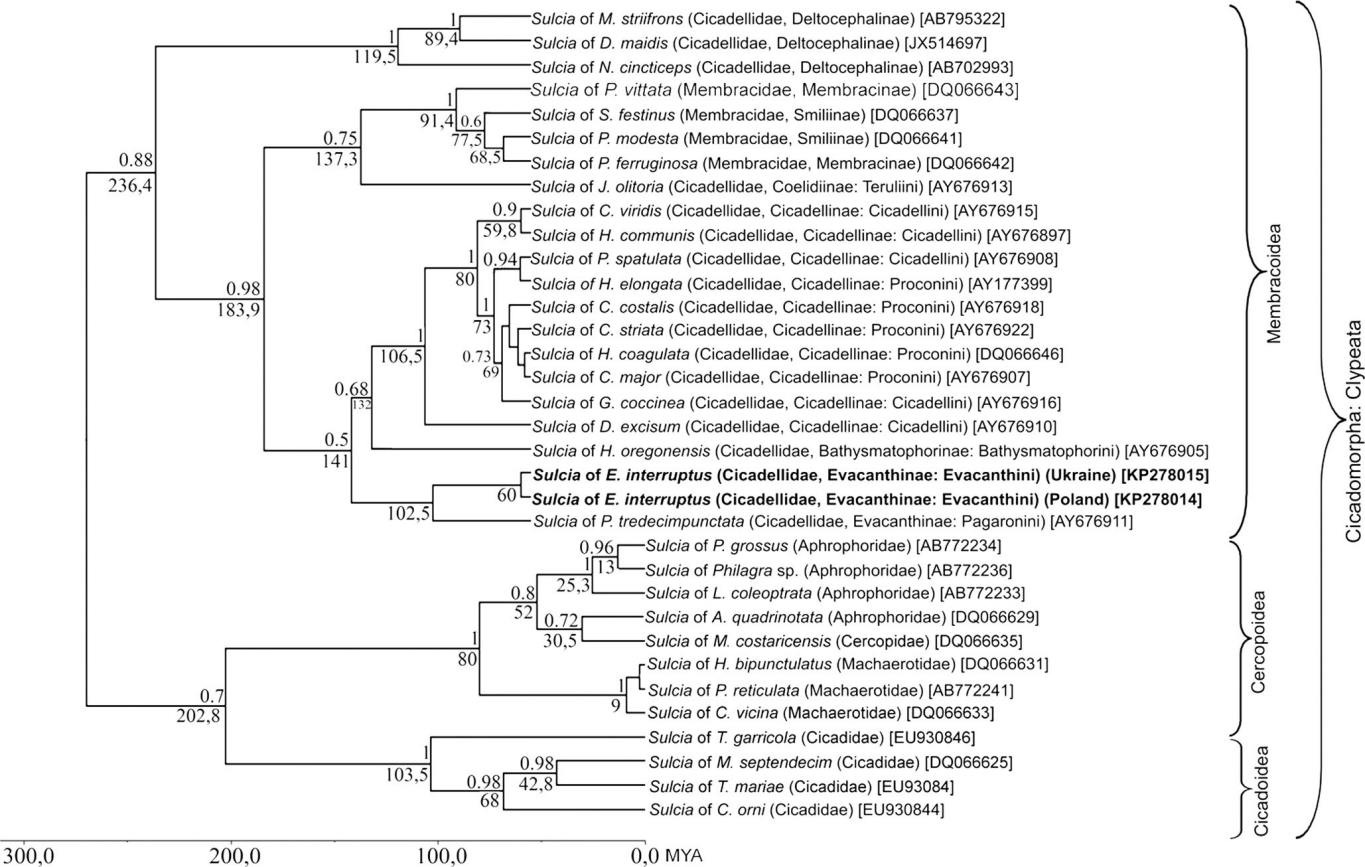
The phylogeny of the *Sulcia* symbionts of the extant Cicadomorpha: Clypeata based on a Bayesian analysis of partial 16S rDNA sequences. Dated tree generated using an uncorrelated longnormal clock in Beast. Posterior probabilities are indicated *above the branches*, whereas the *numbers below the branches* show the time of lineages divergence, scale is given with absolute geological ages (million years ago, *MYA*) (GenBank accession numbers of 16S rRNA genes are given in *brackets*). (Abbreviations for generic names: *A. quadrinotata* – *Aphrophora quadrinotata*; *C. costalis – Cuerna costalis*; *C. major – Cyrtodisca major*; *C. striata – Cuerna striata*; *C. orni – Cicada orni*; *C. vicina – Chaetophyes vicina*; *C. viridis – Cicadella viridis; D. excisum – Diestostemma excisum*; *D. maidis – Dalbulus maidis*; *E. interruptus – Evacanthus interruptus*; *G. coccinea – Graphocephala coccinea*; *H. bipunctulatus – Hindoloides bipunctulatus*; *H. coagulata – Homalodisca coagulata*; *H. communis – Helochara communis*; *H. elongata – Homalodisca elongata*; *H. oregonensis – Hylaius oregonensis*; *J. olitoria – Jikradia olitoria*; *L. coleoptrata – Lepyronia coleoptrata*; *M. costaricensis – Mahanarva costaricensis*; *M. sexnotatus – Macrosteles sexnotatus; M. striifrons – Macrosteles striifrons*; *M. septendecim – Magicicada septendecim*; *M. hiroglyphicus – Matsumuratettix hiroglyphicus; N. cincticeps – Nephotettix cincticeps*; *P. ferruginosa – Philya ferruginosa*; *P. grossus – Ptyelus grossus*; *P. modesta – Publilia modesta*; *P. reticulata – Pectinariophyes reticulata*; *P. spatulata – Pamplona spatulata*; *P. tredecimpunctata – Pagaronia tredecimpunctata*; *P. vittata – Platycotis vittata*; *S. festinus – Spisistilus festinus*; *T. garricola – Tibicina garricola*; *T. mariae – Tettigetta mariae*)

**Fig. 2:**
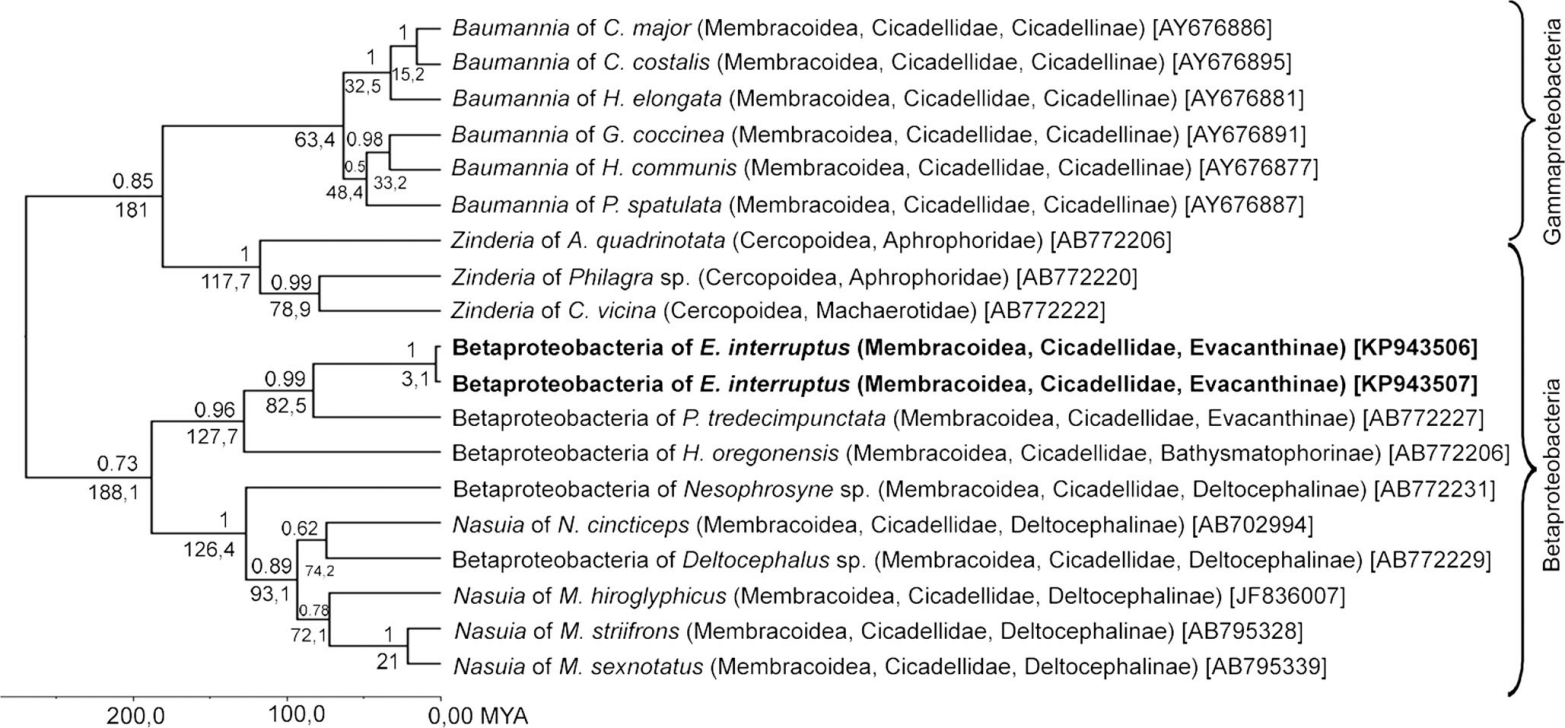
The phylogeny of proteobacterial symbionts from the extant Cicadomorpha: Clypeata based on Bayesian analysis of partial 16S rDNA sequences (abbreviations for generic names as in Fig. 1). Dated tree generated using an uncorrelated longnormal clock in Beast. Posterior probabilities are indicated *above the branches*, whereas the *numbers below the branches* show the time of lineages divergence, scale is given with absolute geological ages (million years ago, *MYA*) (GenBank accession numbers of 16S rRNA genes are given in *brackets*)

The systematic affinity of bacteriome-associated symbionts of *E. interruptus* from both Polish and Ukrainian populations was determined based on the analysis of their 16S rDNA sequences. In both samples (from Poland and Ukraine) the direct PCR revealed the presence of bacteria representing the phylum Bacteroidetes (bacterium *Sulcia*) and class Betaproteobacteria of the phylum Proteobacteria. The specific PCR has not revealed the occurrence of bacterium *Baumannia* in examined species (the specific product was not obtained). A comparison of the obtained 16S rDNA sequences of *Sulcia* symbionts from Polish and Ukrainian populations of *E. interruptus* indicated that they are identical, whereas there is a slight difference between the 16S rDNA sequences of betaproteobacterial symbionts (1 %). The length of the analyzed sequences was 960 pb for *Sulcia* and 678 pb for the betaproteobacterial symbiont, respectively. The mean base compositions of *Sulcia* symbionts were as follows: 30.8 % A, 25.2 % T, 17.3 % C, 26.6 % G, while in the betaproteobacterial symbionts there were: 33.2 % A, 25.9 % T, 15.7 % C, 25.1 % G. The variation within the 16S rDNA was about 12 % variable sites and 9 % parsimony informative sites for *Sulcia* symbionts and about 49 % variable sites and 43 % parsimony informative sites for the betaproteobacteria ones. BLASTN searches against the bacterial 16S rDNA sequences deposited in the GenBank clearly demonstrate the similarity of Bacteroidetes symbiont to the bacterium *Sulcia* isolated from the leafhopper, *Pagaronia tredecimpunctata* (99 %, 1217/1227) [AY676911], whereas the betaproteobacterial symbiont displays the highest similarity to the symbiont found in the two leafhoppers: *Pagaronia tredecimpunctata* (Evacanthinae: Pagaronini Anufriev, 1978) (89 %, 430/480) [AB772227] and *Hylaius oregonensis* (Baker, 1898) (Bathysmatophorinae Anufriev, 1978: Bathysmatophorini Anufriev, 1978) (90 %, 439/491) [AB772226]. The topology resulting from the Bayesian inference of the *Sulcia* and betaproteobacterial symbiont are shown in Figs. 1 and 2, respectively. Phylogenetic analyses of 16S rDNA of *Sulcia* symbionts of various representatives of Cicadomorpha (Fig. 1) show the monophyly of symbionts of the Membracoidea within Cicadomorpha: Clypeata (with 0.88 posterior probability values). Phylogenetic analyses of the 16S rDNA of the betaproteobacterial symbionts indicate that the symbionts of Evacanthinae (*Pagaronia* and *Evacanthus*) and *Hylaius oregonensis* (Bathysmatophorinae) form a monophyletic group which is closely related to betaproteobacterial symbionts of members of the Deltocephalinae leafhoppers (Fig. 2).

Sequence divergences within *Sulcia* symbiont isolated from representatives of Cicadellidae range from 0.1 to 5 % (Table 1), whereas within the betaproteobacterial symbiont from 0.3 to 48.2 % (Table 2).

**Table 1:** Pairwise, genetic distances among 16S rDNA sequences of *Sulcia* symbionts of representatives of Cicadellidae (abbreviations for generic names as in Fig. 1). The values indicate the number of base substitutions. The analysis was conducted using the Jukes-Cantor model

		*Candidatus* Sulcia muellerii																
		1	2	3	4	5	6	7	8	9	10	11	12	13	14	15	16	17
1.	*C. viridis*																	
2.	*C. costalis*	0,002																
3.	*C. striata*	0,001	0,001															
4.	*C. major*	0,001	0,001	0,000														
5.	*D. maidis*	0,043	0,043	0,042	0,042													
6.	*D. excisum*	0,008	0,010	0,009	0,009	0,044												
7.	*E. interruptus* (Poland)	0,014	0,016	0,015	0,015	0,038	0,010											
8.	*E. interruptus* (Ukraine)	0,014	0,016	0,015	0,015	0,038	0,010	0,000										
9.	*G. coccinea*	0,003	0,003	0,002	0,002	0,043	0,010	0,016	0,016									
10.	*H. communis*	0,000	0,002	0,001	0,001	0,043	0,008	0,014	0,014	0,003								
11.	*H. coagulata*	0,001	0,001	0,000	0,000	0,042	0,009	0,015	0,015	0,002	0,001							
12.	*H. elongata*	0,002	0,002	0,001	0,001	0,043	0,010	0,016	0,016	0,003	0,002	0,001						
13.	*J. olitoria*	0,018	0,021	0,019	0,019	0,045	0,019	0,015	0,015	0,021	0,018	0,019	0,019					
14.	*M. striifrons*	0,037	0,037	0,036	0,036	0,008	0,038	0,032	0,032	0,037	0,037	0,036	0,037	0,039				
15.	*N. cinticeps*	0,032	0,034	0,033	0,033	0,012	0,031	0,027	0,027	0,034	0,032	0,033	0,034	0,034	0,007			
16.	*P. tredecimpunctata*	0,012	0,015	0,014	0,014	0,037	0,011	0,006	0,006	0,015	0,012	0,014	0,015	0,017	0,031	0,026		
17.	*P. spatulata*	0,001	0,001	0,000	0,000	0,042	0,009	0,015	0,015	0,002	0,001	0,000	0,001	0,019	0,036	0,033	0,014	
18.	*H. oregonensis*	0,024	0,025	0,025	0,025	0,050	0,021	0,018	0,018	0,026	0,024	0,025	0,026	0,026	0,045	0,040	0,018	0,025

**Table 2:** Pairwise, genetic distances among 16S rDNA sequences of proteobacterial symbionts of representatives of Cicadellidae (abbreviations for generic names as in Fig. 1). The values indicate the number of base substitutions. The analysis was conducted using the Jukes-Cantor model

		Betaproteobacterial symbionts														
		1	2	3	4	5	6	7	8	9	10	11	12	13	14	15
1.	*C. costalis*															
2.	*C. major*	0.022														
3.	*G. coccinea*	0.078	0.076													
4.	*H. communis*	0.066	0.058	0.053												
5.	*H. elongata*	0.030	0.039	0.074	0.068											
6.	*H. oregonensis*	0.308	0.317	0.308	0.296	0.322										
7.	*M. striifrons*	0.473	0.482	0.476	0.479	0.467	0.282									
8.	*N. cinticeps*	0.459	0.461	0.467	0.450	0.436	0.252	0.134								
9.	*P. tredecimpunctata*	0.312	0.322	0.310	0.312	0.322	0.088	0.260	0.239							
10.	*P. spatulata*	0.063	0.069	0.064	0.053	0.054	0.317	0.453	0.439	0.303						
11.	*M. hiroglyphicus*	0.428	0.436	0.431	0.417	0.420	0.274	0.129	0.123	0.254	0.401					
12.	*Nesophrosyne* sp.	0.423	0.423	0.409	0.417	0.415	0.252	0.192	0.161	0.252	0.407	0.153				
13.	*Deltocephalus* sp.	0.461	0.459	0.482	0.461	0.445	0.276	0.153	0.123	0.258	0.450	0.144	0.174			
14.	*M. sexnotatus*	0.447	0.456	0.456	0.459	0.442	0.267	0.035	0.125	0.258	0.445	0.136	0.190	0.153		
15.	*E. interruptus (Poland)*	0.343	0.353	0.341	0.346	0.358	0.125	0.260	0.226	0.102	0.341	0.247	0.237	0.267	0.252	
16.	*E. interruptus (Ukraine)*	0.341	0.351	0.338	0.343	0.355	0.122	0.256	0.222	0.099	0.338	0.243	0.233	0.263	0.247	0.003

### Ultrastructure, distribution and transovarial transmission of symbionts

**Fig. 3:**
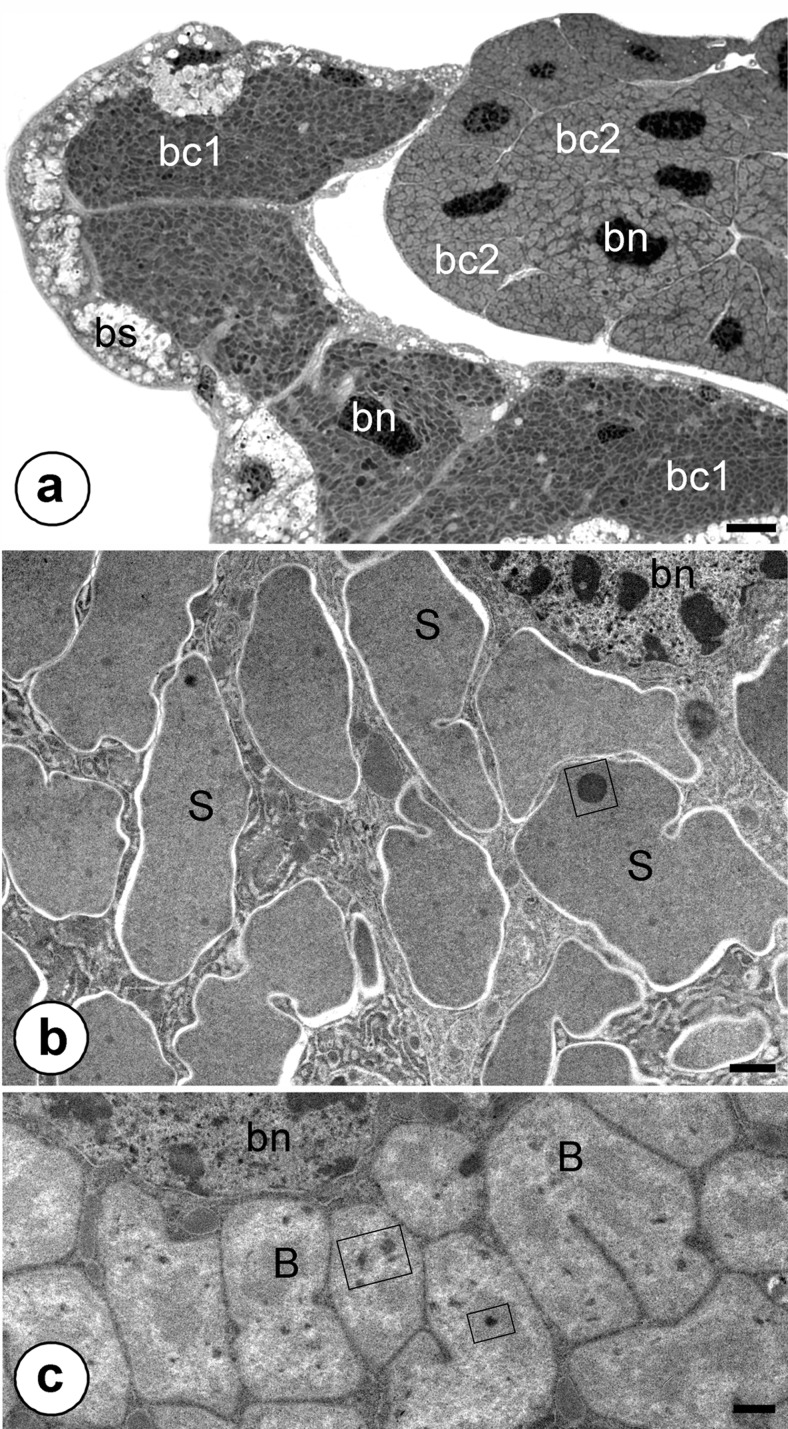
Organization of the bacteriomes of *Evacanthus interruptus*. **a** Fragment of the bacteriome composed of bacteriocytes containing the bacterium *Sulcia* (*bc1*) and bacteriocytes containing betaproteobacterial symbiont (*bc2*). Bacteriocyte nucleus (*bn*), bacteriome sheath (bs). Methylene blue, *scale bar* = 20 μm. **b** Fragment of the bacteriocyte containing the bacterium *Sulcia* (*S*). Bacteriocyte nucleus (*bn*), electron-dense granule in the cytoplasm of *Sulcia* (*in frame*). TEM, *scale bar* = 2 μm. **c** Fragment of the bacteriocyte containing the betaproteobacterial symbiont (*B*). Bacteriocyte nucleus (*bn*), electron-dense granule in the cytoplasm of the betaproteobacterial symbiont (*in frame*). TEM, *scale bar* = 2 μm

**Fig. 4:**
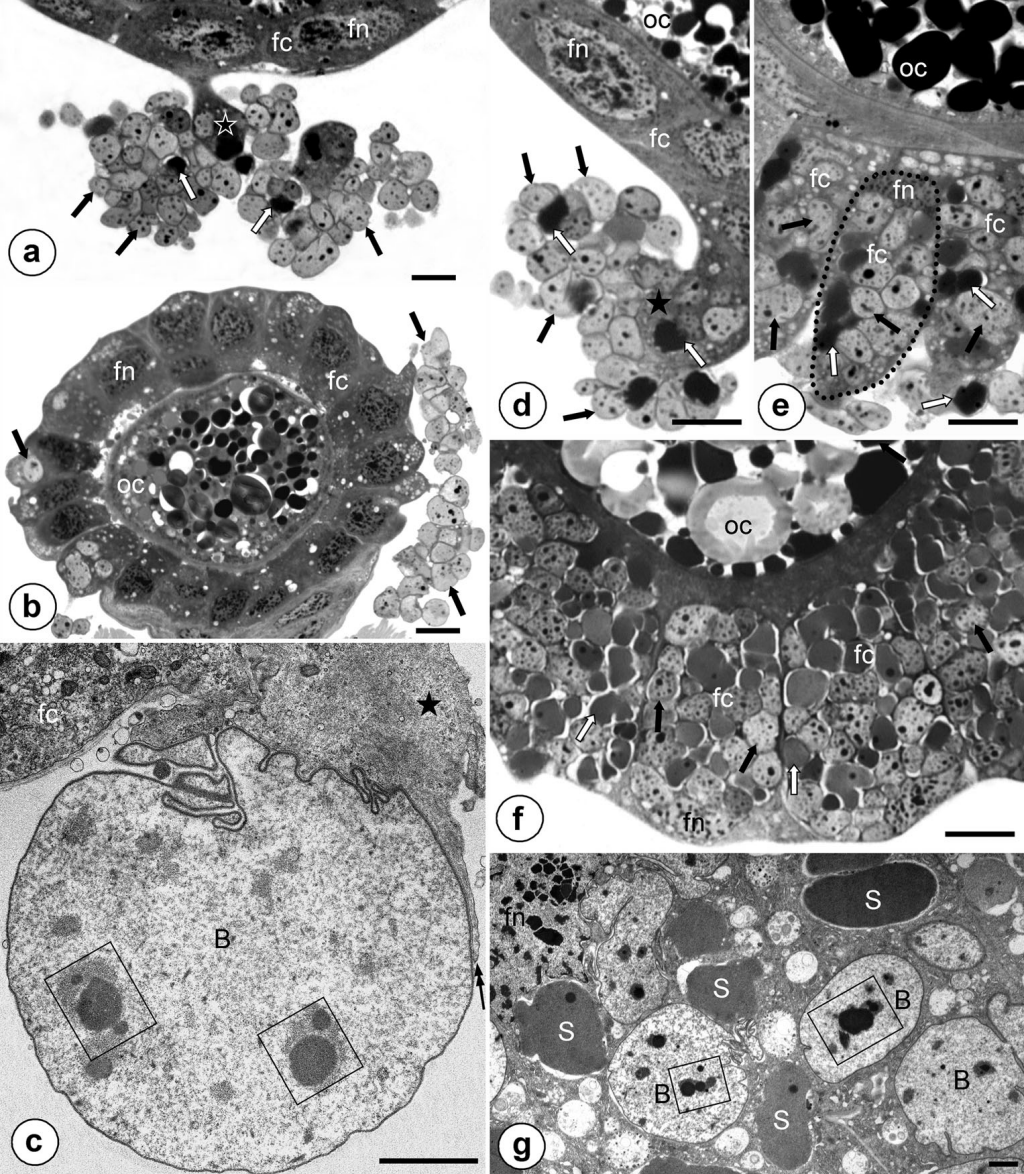
Transovarial transmission of symbionts. **a**, **b** Symbiotic bacteria (*Sulcia* - *white arrows*, betaproteobacterial symbionts - *black arrows*) that gather around the posterior pole of the terminal oocyte (*oc*) and start to enter cytoplasm of follicular cells (*fc*). Projection of the follicular cell (*black asterisk*), nucleus of follicular cell (*fn*). Methylene blue, *scale bar* = 20 μm. **c** Betaproteobacterial symbiont (*B*) enters (*double black arrow*) the projection (*black asterisk*) of the follicular cell (*fc*). Electron-dense granule in the cytoplasm of the betaproteobacterial symbiont (*in frame*). TEM, *scale bar* = 2 μm. **d** Fragment of the terminal oocyte (*oc*) (*longitudinal section*). Note bacteria *Sulcia* (*white arrows*) and betaproteobacterial symbionts (*black arrows*) that via massive projection (*black asterisk*) enter cytoplasm of follicular cells (*fc*). Nucleus of follicular cell (*fn*). Methylene blue, *scale bar* = 20 μm. **e** Fragment of the terminal oocyte (*oc*) (*longitudinal section*). Note enlarged follicular cells (*fc*) filled with symbiotic bacteria (*encircled with dotted line*) and symbiotic bacteria (*Sulcia - white arrows*, betaproteobacterial symbionts *- black arrows*) that invade follicular cells. Nucleus of follicular cell (*fn*). Methylene blue, *scale bar* = 20 μm. **f** Fragment of the terminal oocyte (*oc*) surrounded by follicular cells (*fc*) that are tightly packed by symbiotic bacteria (*Sulcia - white arrows*, betaproteobacterial symbionts *- black arrows*) (*cross section*). Nucleus of follicular cell (*fn*). Methylene blue, *scale bar* = 20 μm. **g** Bacteria *Sulcia* (*S*) and betaproteobacterial symbionts (*B*) in the cytoplasm of follicular cell. Nucleus of follicular cell (*fn*), electron-dense granules in the cytoplasm of the symbionts (*in frames*). TEM, *scale bar* = 2 μm

**Fig. 5:**
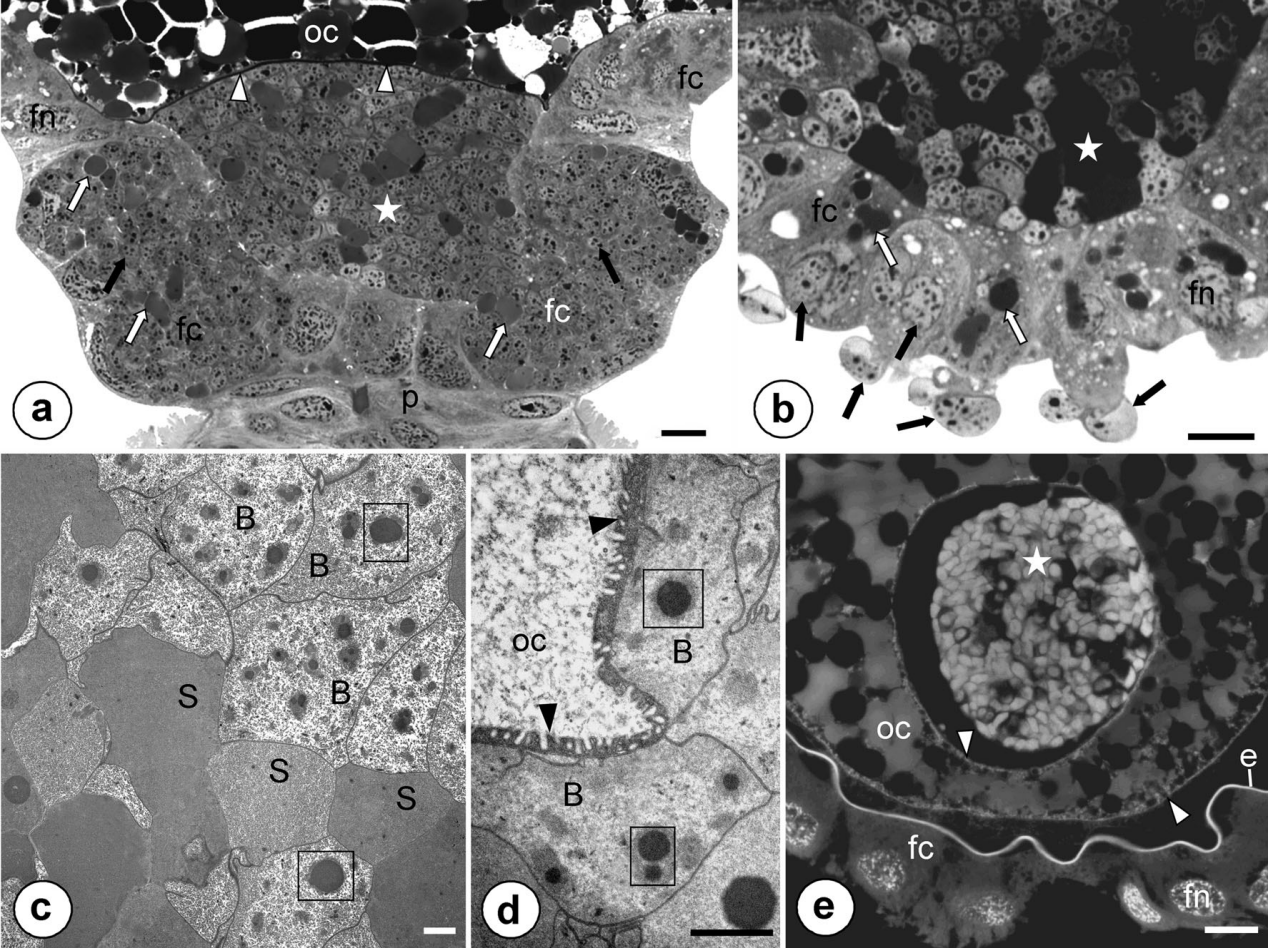
Transovarial transmission of symbionts. **a** Fragment of the terminal oocyte (*oc*) (*longitudinal section*). Note the follicular cells (*fc*) filled with symbiotic bacteria (*Sulcia - white arrows*, betaproteobacterial symbionts *- black arrows*) and accumulation of symbionts in the perivitelline space (*white asterisk*). Nucleus of follicular cell (*fn*), ovariolar stalk (*pedicel*) connecting the ovariole to the lateral oviduct (*p*), oolemma (*arrowhead*). Methylene blue, *scale bar* = 20 μm. **b** Fragment of the terminal oocyte during the late stage of infectioning by bacteria (*cross section*). Note the projections of follicular cells (*fc*) invaded by bacteria (*Sulcia - white arrows*, betaproteobacterial symbionts *- black arrows*) and the accumulation of symbionts in the perivitelline space (*white asterisk*). Nucleus of follicular cell (*fn*). Methylene blue, *scale bar* = 20 μm. **c** and **d** The accumulation of symbionts in the perivitelline space. Bacterium *Sulcia* (*S*), betaproteobacterial symbionts (*B*), oocyte (*oc*), oolemma (*arrowhead*), electron-dense granules in the cytoplasm of symbionts (*in frames*). TEM, *scale bar*= 2 μm. **e** The posterior pole of the oocyte (*oc*) (*cross section*). Note a characteristic ‘symbiont ball’(*white asterisk*) in the deep invagination of the oolemma (*arrowhead*). Follicular cells (*fc*), nucleus of follicular cell (*fn*), egg envelope (*e*). Fluorescence, confocal microscope, DAPI staining, *scale bar* = 20 μm

The ovaries of *Evacanthus interruptus* are accompanied by large, paired, yellow-colored bacteriomes that are composed of two well-defined parts (Fig. 3a): (1) the part located in the close neighborhood to the body wall that is surrounded by a single layer of epithelial cells (bacteriome sheath) and (2) the part that adheres to the ovaries. Both parts of the bacteriomes consist of numerous giant bacteriocytes (Fig. 3a) which are tightly packed with morphologically distinct bacteria (Fig. 3b, c). Ultrastructural observations revealed that bacteriocytes occupying part of the bacteriome adhering to the body wall contain large, electron-dense, pleomorphic bacteria (Fig. 3b), whereas in bacteriocytes adhering to the ovaries there are large, pleomorphic, electron-translucent bacteria (Fig. 3c). The cytoplasm of the latter is more heterogenous (Fig. 3b) than the cytoplasm of the electron–dense bacteria (Fig. 3c). Both bacteria are characterized by the presence of electron-dense granules in their cytoplasm (Figs. 3b, c, 4c, g and 5c, d). The comparison of the results of morphological observations and results of molecular identification using specific pairs of primers amplifying 16S rRNA gene indicated that electron–dense, pleomorphic bacteria belong to the Bacteroidetes bacterium *Sulcia*, whereas electron–translucent bacteria represent the betaproteobacterial symbiont. Ultrastructural studies have not revealed the presence of bacteria in the bacteriome sheath.

The symbionts are transmitted between generations transovarially, i.e. by means of the infection of oocytes. In the mature females which possess terminal oocytes at the stage of advanced vitellogenesis (for the characteristics of ovaries and process of oogenesis of Cicadomorpha see Książkiewicz-Kapralska 1985), both types of bacteria leave the cytoplasm of the bacteriocytes and begin to gather around the terminal oocytes (Fig. 4a, b, d). Then, the symbionts enter the cytoplasm of follicular cells which form a single-layered epithelium around the oocytes (Fig. 4a, b, d, e). During the bacterial invasion, the follicular cells surrounding the posterior pole of each terminal oocyte form massive projections (Fig. 4a, c, d) which enable the entry of these microorganisms into their cytoplasm. As the bacteria gradually accumulate into the cytoplasm, the volume of follicular cells markedly increases (Fig. 4e, f). As a result, the cytoplasm of the follicular cells is tightly packed with symbiotic microorganisms of both types (Fig. 4f, g). Next, the symbionts are gradually released into the perivitelline space (i.e. the space between the oocyte and follicular epithelium), where they assemble in the deep depression of the oolemma (Fig. 5a, b), forming finally a characteristic ‘symbiont ball’ (Fig. 5c, e). Up until the end of the oogenesis process (i.e. oocyte growth) the bacteria do not invade the ooplasm (i.e. they are isolated from the ooplasm by oolemma) (Fig. 5d, e). When the formation of the ‘symbiont ball’ has been finalized, the follicular cells surrounding the posterior pole of the oocyte begin synthesizing with the egg envelopes (Fig. 5e).

## Discussion

Our observations revealed that two types of symbiotic bacteria are harbored in the body of *Evacanthus interruptus* (Evacanthinae: Evacanthini): Bacteroidetes symbionts *Sulcia* and betaproteobacterial symbiont closely related to the symbiont found in two other representatives of Cicadellidae: *Pagaronia tredecimpunctata* (Evacanthinae: Pagaronini) and *Hylaius oregonensis* (Bathysmatophorinae: Bathysmatophorini). These findings could suggest closer or more ancient relationships of these subfamilies. Dietrich (1999) considered Pagaronini and Evacanthini to be closely related, later (Dietrich 2004) united them together with Nirvanini in the subfamily Evacanthinae. However, available data (Dietrich 1999; Dietrich et al. 2001, 2005), provide no evidence of a close relationship of Evacanthinae to Bathysmatophorinae (Dietrich 2004; Wei et al. 2010). Known fossils cannot elucidate that relationship – the oldest Evacanthinae (Nirvanini) were reported from the Miocene Dominican amber (Dietrich and Vega 1995), while Bathysmatophorinae are more ancient, with fossil record reaching the late Cretaceous period (Szwedo 2005). The presence of the Bacteroidetes symbiont *Sulcia* and betaproteobacterial symbiont seems to be a very ancient and conservative heritage among Cicadellidae.

On the other hand, *Evacanthus interruptus*, *Pagaronia tredecimpunctata* and *Hylaius oregonensis* retained the ancestral betaproteobacterial symbiont which forms a well-defined clade with betaproteobacterial symbionts living in the members of the Deltocephalinae leafhoppers (see Fig. 2). These findings are consistent with the conclusion reached from the molecular data analysis indicating that betaproteobacterial symbionts are descendants of a bacterium which infected the shared ancestor of leafhoppers and spittlebugs (Bennett and Moran 2013; Koga et al. 2013; Koga and Moran 2014; Bennett et al. 2014). Recent molecular studies have also revealed that during the evolution of some lineages of the Cicadomorpha: Clypeata, the betaproteobacterium has been replaced by other bacteria (e.g. *Baumannia*, *Hodgkinia*, *Sodalis*-like bacteria *–* see Introduction). This more recent infection could be as ancient as the separation time of Cicadomorpha: Clypeata evolutionary lineage in the early Triassic period from the other Cicadomorpha (ancestral Hylicelloidea, and not directly related Palaeontinoidea and Dysmorphoptiloidea), which retained ancestral phloem-feeding (Wang et al. 2012), or even older. On the other hand, Shcherbakov (2012) stated that ‘leafhopperization’, i.e. the successive acquisition of membracoid characters leading to the modern Cicadellidae lineage, was a long process that spanned about a hundred million years, during the Jurassic and Cretaceous periods. Modern Cicadellidae diversity is definitively the result of their most recent radiation and, as for numerous other phytophagous groups, the effect of the Mid-Cretaceous biotic crisis and reorganization of the biosphere and later Cenozoic habitat diversification (Szwedo 2008). The oldest Euhemiptera (a unit comprising of Fulgoromorpha, Cicadomorpha, Coleorrhyncha and Heteroptera) have been reported from Late Carboniferous (Nel et al. 2013), so the infection with Bacteroidetes symbionts *Sulcia* and the betaproteobacterial symbiont could be more ancient (lost in Coleorrhyncha and Heteroptera). It took place probably more than 300 million years ago, maybe in relation to late Carboniferous rainforest collapse (DiMichele et al. 2010; Sahney et al. 2010; Cascales-Miñana and Cleal 2013).

Our studies did not revealed the presence in the body of *E. interrupus* of bacterium *Baumannia* that is characteristic for most members of Cicadellidae: Cicadellinae *s. str.* (Moran et al. 2003; Takiya et al. 2006; Wu et al. 2006; Bennett et al. 2014). This absence of *Baumannia* supports placement of Evacanthini in separate subfamily (Metcalf 1963; Dietrich 2004), and more remote relationships with Cicadellinae *s. str*., to which the tribe Evacanthini was formerly subordinated (Hamilton 1983; Oman et al. 1990), where Cicadellinae was treated *sensu lato*.

Earlier and more recent observations (Buchner 1925, 1965; Cheung and Purcell 1999; Noda et al. 2012; this study) indicate that of all of the leafhoppers examined thus far, the obligate symbionts are localized in the cytoplasm of separate bacteriocytes which are integrated into large organs termed bacteriomes. It seemed, thus, that in spite of the presence of diverse types of symbionts, the distribution of symbionts in the body of leafhoppers is similar. However, what has recently appeared is that in the green leafhopper, *Cicadella viridis*, its symbionts (i.e. *Sulcia* and *Sodalis*-like symbionts) may occur in separate bacteriocytes or may coexist in the same bacteriocyte (Michalik et al. 2014a). Moreover, it has been observed that in the common bacteriocytes *Sodalis*-like symbionts invade the cells of *Sulcia*, resulting in the occurrence in the cytoplasm of these bacteriocytes bacterium *Sulcia* and *Sodalis*-like symbionts inside the cells of *Sulcia*. Based on this observation, Michalik and co-workers (2014a) hypothesized that the symbiosis in *Cicadella viridis* represents the youngest state of association (i.e. the beginning of acquisition of *Sodalis*-like symbionts by *Sulcia*). In turn, in *Macrosteles laevis* (Kobiałka et al. 2014), some cells of *Sulcia* contain small rod-shaped bacteria, but the latter never occur individually. Thus, it seems that the distribution of symbionts and the state of symbiosis in members of the Cicadellidae is more complex than previously supposed.

Both earlier observations with the use of light microscopy (Buchner 1925, 1965; Müller 1962) and more recent ultrastructural studies (e.g. Cheung and Purcell 1999; Szklarzewicz and Moskal 2001; Szklarzewicz et al. 2006, 2013; Sacchi et al. 2008; Michalik et al. 2013; Michalik et al. 2014a, b; Kobiałka et al. 2014; Swiatoniowska et al. 2013; this study) have shown that in hemipterans, mycetomic symbionts may be transmitted from mother to the offspring in a different way, e.g. they may invade larval ovaries with undifferentiated germ cells (in some scale insects) or oocytes in adult females (in most hemipterans), they may be released from bacteriocytes and individually invade ovaries (in most hemipterans) or whole intact bacteriocytes may enter ovaries (in whiteflies). It should be stressed that even in closely related groups of hemipterans (e.g. within some families of scale insects), the transmission of symbionts may take a different course. Both earlier and recent studies (Buchner 1925, 1965; Müller 1962; Cheung and Purcell 1999; Sacchi et al. 2008; Michalik et al. 2014a; Kobiałka et al. 2014; this study) indicate that in Cicadomorpha, the symbionts infect the ovaries via follicular cells surrounding the posterior pole of the oocyte. Thus, in contrast to other hemipterans, all the lineages of Cicadomorpha: Clypeata developed the similar mechanism of transmission of symbionts from one generation to the next.
